# MultiShapeC, an algorithm to assess concentration in multi-shape nanoparticle samples: nanosilver, a case study†

**DOI:** 10.1039/d2ra04078f

**Published:** 2022-09-20

**Authors:** Rodrigo Nicolás Núñez, Alicia Viviana Veglia, Natalia Lorena Pacioni

**Affiliations:** Universidad Nacional de Córdoba, Facultad de Ciencias Químicas, Departamento de Química Orgánica Haya de la Torre y Medina Allende s/n, Ciudad Universitaria X5000HUA Córdoba Argentina nataliap@fcq.unc.edu.ar n.lpacioni@unc.edu.ar +54-351-5353867 ext. 55374; Consejo Nacional de Investigaciones Científicas y Técnicas (CONICET), INFIQC Córdoba Argentina

## Abstract

Shape, size, and dispersity play a crucial role in the calculation of colloidal nanoparticle concentrations, which results in remarkable differences in the determination of parameters like Stern–Volmer constants. In this work, we propose an algorithm named MultiShapeC to include the variability in shapes and polydispersity in the concentration calculation. This algorithm was validated using the quenching of carbazole fluorescence emission by silver nanoparticles.

## Abstract 2

An algorithm to include multi-shape and polydispersity in the nanoparticle concentration calculation is presented.

## Graphical abstract

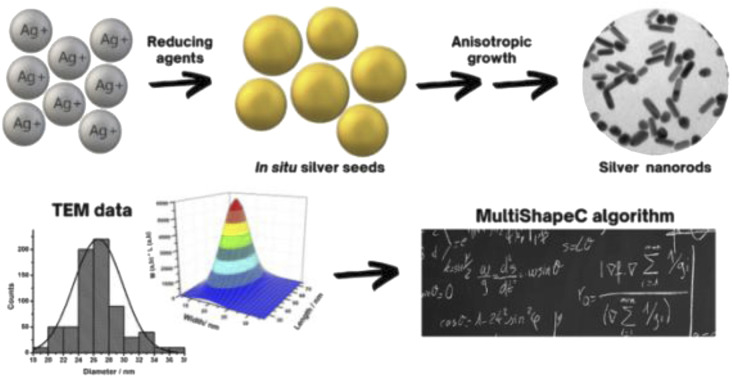

## Introduction

Engineered nanomaterials are undoubtedly relevant to different research areas. Since their discovery, these materials have shown many intriguing physicochemical properties leading to several applications in catalysis,^1–4^ biomedicine,^5–9^ sensing,^10–15^ electronics,^16–18^ and optics.^19–23^

Anisotropic metallic particles, such as silver and gold nanorods, caught a particular interest for light-related applications since they exhibit two plasmon resonance bands.^24,25^ These extinction bands are the longitudinal surface plasmon resonance (LSPR), related to electron oscillations along the particle's length, and the transverse one (TSPR), corresponding to electron oscillations along their width. The position of LSPR and TSPR can be finely tuned by controlling the aspect ratio of the particles.^26,27^

In the case of silver nanorods (AgNR), synthetic protocols usually involve both chemical^28–33^ and physical methods.^34^ However, these methodologies require templates as directors to control the shape of the resulting nanoparticles.^28,30,31,33^ One of the most common synthetic routes for obtaining AgNR is the seed-mediated growth method.^28,31,35,36^ This procedure comprises seed nanoparticles that are added to a growth solution containing cetyltrimethylammonium bromide (CTAB) – a cationic surfactant – and a mild reductant like ascorbic acid. CTAB forms rod-shaped micelles in aqueous solutions that act as soft templates to shape the particles.^28,36–38^ While most of those syntheses effectively control the shape, dimension, and yield of the particles, removing the surfactants from the surface would entail harsh post-synthesis treatments, thus making applications that demand analyte adsorption on the surface of the nanoparticle an arduous task.

Characterization of nanomaterials is essential to obtain reliable information about effects attributed to the nanoparticle and avoid misleading results. The accurate determination of their concentration is one of the most critical parameters, mainly because nanoparticles (NP) are frequently obtained with a certain degree of polydispersity,^39^ indeed when it is possible to get highly uniform NP, for example, nanospheres.^40,41^ A few publications discuss this major challenge in nanoscience, focusing on polydispersity.^42,43^ However, the calculation of NP concentration in multi-shape samples remains generally underestimated due to overlooking each shape's contribution to the total NP concentration. To overcome this limitation, in this work, we present Multiple Shape Corrector (MultiShapeC), an algorithm to estimate the total concentration of NP more accurately by also considering the NP proportions by morphology and size.

To accomplish this goal, we chose silver nanoparticles as the case study. First, we developed an experimental protocol to obtain multi-shaped AgNP in a controllable manner through a seedless and surfactant-less synthetic methodology. The proposed method consists of a one-pot, two-sequential step process. The first step, occurring at lower temperatures, leads to the *in situ* formation of the silver nanospheres nuclei that then grow as rod-shaped particles during a second step at higher temperatures due to the addition of a strictly controlled amount of sodium hydroxide. Once the nanoparticles were characterized, we used MultiShapeC to calculate the total NP concentration. Finally, as a case study, we compared the impact of the AgNP concentration calculation (MultiShapeC *vs.* the average size method) on the determination of Stern–Volmer constants.

## Experimental

### Instrumentation

Absorption spectra were obtained using quartz cells (1 cm path-length) in a UV-vis Shimadzu 1800 spectrophotometer working in the (200–800) nm wavelength range. The pH was controlled using an Orion (Boston, MA, USA) model 720A pH-meter with a Ross combination pH electrode. Previously, the pH meter was calibrated with standard buffers at pH = 4.008, 6.994, and 9.136. An Eppendorf Centrifuge 5804 was employed for centrifugation, and a TEM-Jeol 1120 electron microscope, 80 kV accelerating voltage, was used for obtaining Transmission Electron Microscopy (TEM) images. Fluorescence measurements were done using a Cary Eclipse fluorescence spectrophotometer (Agilent) with a Peltier temperature controller set at 25.0 °C. Data analysis was performed using Origin 8.0®.

### Materials and methods

Silver nitrate ≥99% (Biopack), sodium borohydride (Tetrahedron), trisodium citrate (Anedra), sodium hydroxide (Cicarelli), sodium acetate (Anedra), acetic acid (Cicarelli), 3,3′,5,5′-tetramethylbenzidine ≥99% (TMB, Aldrich) and carbazole (CZL, Sigma) were all analytical grade and used as received. All experiments were conducted in water, Milli-Q quality (resistivity, 25 °C: 18.2 MΩ cm).

#### Synthesis of silver nanoparticles

In a double-neck round bottom flask, 1.00 mM NaBH_4_ and 1.06 mM sodium citrate were mixed (50.0 mL). Then, this solution was heated at 60.0 °C for 30 minutes in the dark and with magnetic stirring at medium speed. Afterward, a AgNO_3_ solution was added dropwise under continuous stirring to the mixture until reaching a 1.0 mM concentration. Then, the temperature was raised to 90.0 °C before adding 0.9 mL of 0.1 M NaOH. The heating continued for 20 minutes more. Next, the colloidal suspension was centrifuged three times at increasing relative centrifugal forces (rcf) to remove unreacted reagents, pre-concentrate the particles, and partially separate spheres from rod-shaped nanoparticles. The centrifugation protocol was 1159 rcf, 2608 rcf, and 15 585 rcf for 30 min, 20 min, and 15 min, respectively, followed by redispersion in deionized water and stored at 4 °C for further use.

#### Characterization

First, the UV-visible spectra were recorded to assess the position of the LSPR and TSPR bands. Then, particle shape and size distributions were determined using TEM images of AgNP colloidal solutions delivered onto carbon-coated copper grids (300 mesh, Electron Microscopy) before drying under air. Image analysis was performed using ImageJ® software and counting around 1000–1500 particles.

#### Quantification of unreacted Ag^+^

Quantification of unreacted Ag^+^ was performed according to the protocol described by Gonzalez-Fuenzalida *et al.*^44^ Five samples containing a fixed amount of AgNP spiked with standard Ag^+^ solutions in the 0–100 mM concentration range were prepared by duplicate. Then, 0.66 mM of TMB in acetate buffer pH 4.00 was added, and after reacting for 15 min, the absorption spectrum was recorded.

### MultiShapeC algorithm for concentration calculation in multi-shaped nanoparticle samples

Based on the work by Lazurko *et al.*,^42^ a modified algorithm is proposed for the estimation of nanoparticle concentration in polydisperse and multi-shaped systems. For example, in a colloidal dispersion of spherical nanoparticles (NS), the total concentration of any given NP system ([NS]) satisfies the equation

1 [NS]∫*F*(*s*)*g*(*s*)d*s* = [*S*]

where *F*(*s*) denotes the total number of atoms for a nanoparticle of diameter “*s*,” *g*(*s*) is a function that describes the size distribution of the nanoparticle system, and [*S*] is the total concentration of nanoparticle precursor used for the fabrication of the NS.^45^ In a multi-shaped system of non-interacting particles, a new term is added to the equation to account for the contribution of nanorods (NR) to the total concentration of particles, resulting in the equation

2 [NS]∫*F*(*s*)*g*(*s*)d*s* + [NR]∫∫*M*(*a*,*b*)*L*(*a*,*b*)d*a*d*b*

where *M* is a function that describes the total number of precursor atoms on a rod nanoparticle of diameter “*a*” and length “*b*,” and *L* is a function that represents the size distribution of rods both in diameter and in length.

Then, using the Riemann sum and the Trapezoidal rule to approximate the integrals, we obtain the equation3
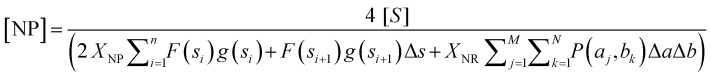
where [NP] is the total concentration of particles, *X*_NP_ and *X*_NR_ are the relative ratios of NS and NR, respectively, in the multi-shaped system, and *P*(*a*,*b*) is a four terms equation that includes the approximated functions *M*(*a*,*b*) and *L*(*a*,*b*). This expression allows the calculation of the concentration of any given NP system independently of the shape and the size distribution of the particles.

The number of precursor atoms in any nanoparticle of specific dimensions, *F*(*s*) and *M*(*a*,*b*), can be calculated using the volume of the particles and atoms according to the equation,4
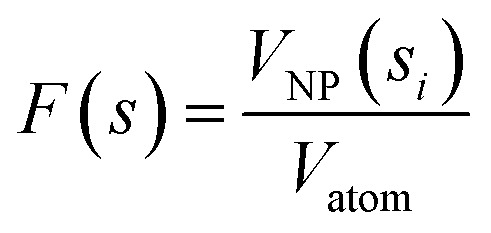
and the equation5
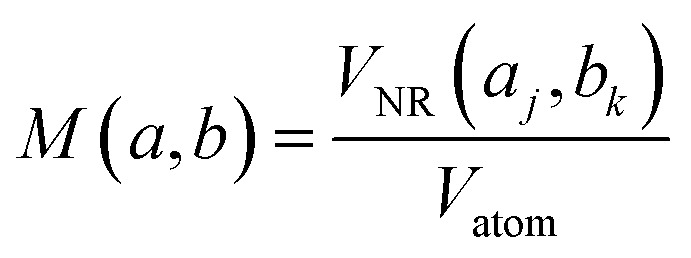
respectively, where *V*_NP_ and *V*_NR_ are the volumes of spherical and rod-shaped nanoparticles, correspondingly, and *V*_atom_ is the volume of a precursor atom. Then, the functions *g*(*s*) and *L*(*a*,*b*) represent the size distribution of NS and NR obtained, for example, through TEM images.

The MultiShapeC algorithm has been incorporated in a fillable spreadsheet for gold and silver nanospheres and nanorods, available as additional material (see the ESI† for the tutorial on how to use it).

In order to evaluate the impact of using MultiShapeC *vs.* the average size method for the NP concentration calculation, we studied the interaction between AgNP and carbazole by fluorescence quenching.^40^ Different aliquots of AgNR–AgNS (0–1.35 nM) were mixed with a fixed concentration of carbazole (100 nM), and the fluorescence emission spectra of the mixtures were recorded between 350 nm and 600 nm. Fluorescence intensities were corrected as in our previous work by considering inner filter effects.^40^

## Results and discussion

### Synthesis and characterization of silver nanoparticles

**Fig. 1:**
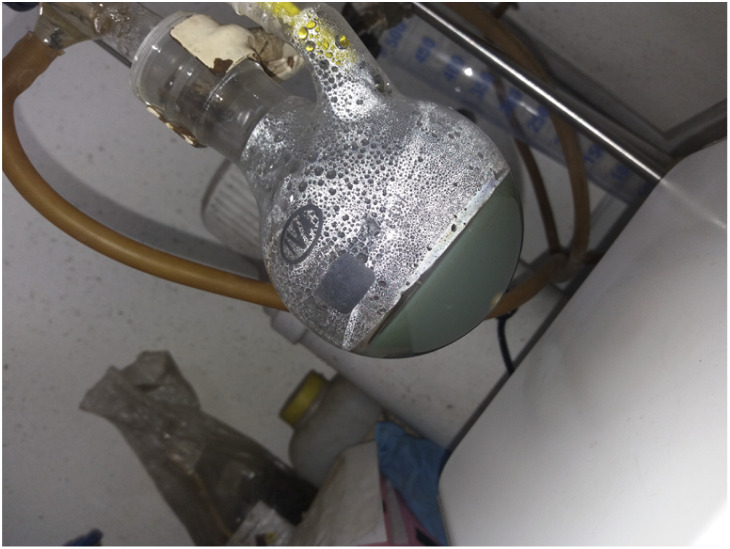
Picture showing the appearance of the reaction crude after the addition of NaOH, and temperature increase to 90 °C.

Spheres and rods AgNP were synthesized using a one-pot and sequential two reductants methodology inspired by Caswell *et al.*^46^ and Agnihotri *et al.*^47^ Initially, sodium borohydride reduced Ag^+^ at 60 °C, followed by a second reduction step at 90 °C mainly due to sodium citrate which also plays an essential role as the stabilizing agent. During the first reduction, the mixture of reactants became deep yellow as AgNS formed. Then, after adding sodium hydroxide (pH > 11) and increasing the temperature, the solution took a green-grayish appearance, as shown in Fig. 1. In this second step, the previously formed Ag^0^ nuclei act as seeds for the growth of nanorods resulting from the reduction of Ag^+^ excess by the citrate anion at high temperatures.^46^

**Fig. 2:**
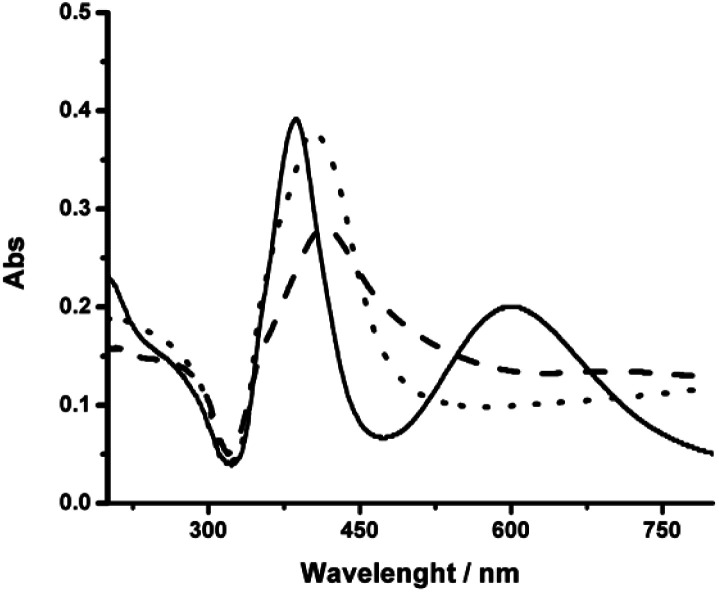
UV-vis spectra of nanosilver colloidal dispersions after synthesis at different NaOH concentrations (mM): 1 (----), 1.8 (—) and 3 (– –). The spectra were measured after centrifugation and resuspension in water at 25.0 °C.

After the centrifugation process, the resuspended pellet presented a green color, attributed to the presence of AgNR, while the supernatant remained yellow due to the presence of nanospheres. The UV-vis spectrum presented the two bands corresponding to the transversal (higher energy absorption) and longitudinal (lower energy absorption) surface plasmons resonance (SPR) modes, characteristic of AgNR (Fig. 2).^24,25^

Factors like reactant's molar ratio, pH, time, and temperature were screened to find the optimal conditions for synthesizing the bi-shaped samples of AgNP. Temperature over 95 °C, hydroxide concentration higher than 0.1 M, and citrate near 8 mM, all lead to a precipitate on the flask walls, attributed to the high activity of citrate as a reductant under these conditions.^48^

**Fig. 3:**
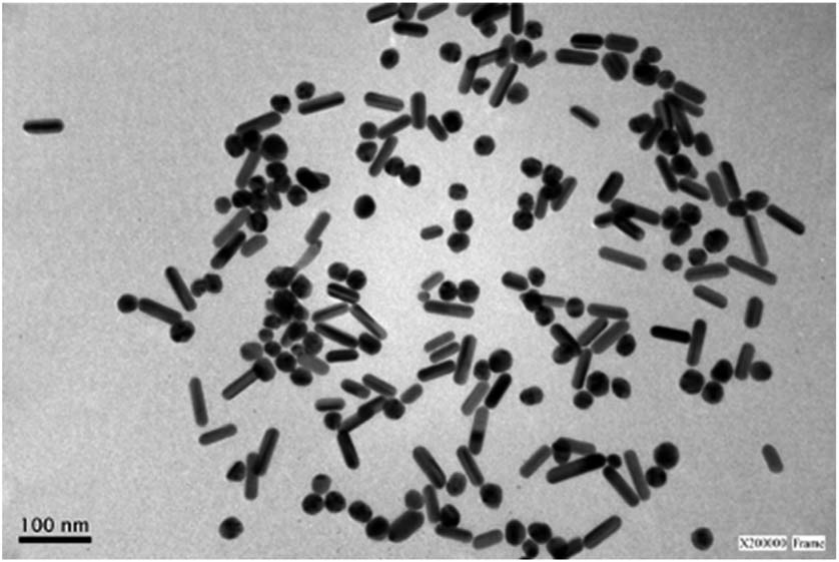
Representative TEM image of a sample of silver nanoparticles after centrifugation showing the presence of rods and spheres. See Fig. S3† for size distribution histograms.

Then, the formation of AgNR was evaluated at different NaOH concentrations, maintaining all other experimental conditions constant. Fig. 2 shows the UV-vis spectra of silver particles formed using NaOH concentration from 1 mM to 3 mM. Above 3 mM, irreversible particle flocculation was always observed. Remarkably, at 1.8 mM NaOH (pH ∼ 11.25), the colloidal dispersion presented two extinction bands at 387 nm and 602 nm, respectively (Fig. 2), in agreement with the expected transversal and longitudinal SPR for AgNR.^35,49^ The presence of NR was confirmed by TEM (Fig. 3). When the hydroxide concentration was 1 mM or 3 mM, no longitudinal SPR appeared, and much of the extinction above 500 nm was ascribed to light scattering from large nanoparticle agglomerates. These results indicated that rod particle formation was highly dependent on the concentration of hydroxide anions during the growth step produced in the second reduction by citrate.

The reaction yield was determined using the spectrophotometric method for the *in situ* quantification of unreacted Ag^+^ in the presence of AgNP.^44^ Thus, using the method of standard additions (MOSA), we found that the amount of unreacted Ag^+^ before centrifugation was below the detection limit (3.5 μM), which means with a 95% confidence level that at least 99.7% of Ag^+^ was reduced to silver particles (see ESI†).

Fig. 3 shows a representative TEM image (after centrifugation) of AgNR obtained using 1.8 mM NaOH. The rod-shaped particles had an average length of 54 nm (standard deviation, sd = 9 nm) and an average width of 18 nm (sd = 2 nm). Also, nanospheres are observed. These AgNS presented an average diameter of 26 nm (sd = 3 nm), close to the nanorods' width (see Fig. S3† in the ESI for size distribution histograms). As the position of the plasmon resonance bands is related to the particle dimensions, the nanospheres absorption band appeared in the same region as the transversal surface plasmon band of the rods. Therefore, the spectra for each type of nanoparticle were overlapped in the 300–450 nm region. The UV-vis spectra displayed a higher absorbance at 387 nm than the band at 603 nm because of the simultaneous interaction of rods and spheres with light. While different approaches were applied to achieve better separation of spherical and rod-shaped nanoparticles after the synthetic procedure (step centrifugation, gravity precipitation), the significant similarity in sizes of both types of particles made the process only partially successful. The best results led to obtaining 47% of spheres and 53% of rods.

Generally, the particle morphology can be explained by the selective ion adsorption like chloride and bromide on facets during the crystal growth.^50,51^ In our case, in a similar fashion, as reported in the synthesis of silver nanowires,^46,52^ citrate and hydroxide anions are also likely to act as shape directors to obtain the nanorods. For example, under similar experimental conditions, Murph *et al.*^52^ found that silver nuclei turned into nanowires due to the oriented coupling phenomena.^53^

Although our results do not discriminate the mechanism for rod formation, the synthetic methodology is reproducible.

**Fig. 4:**
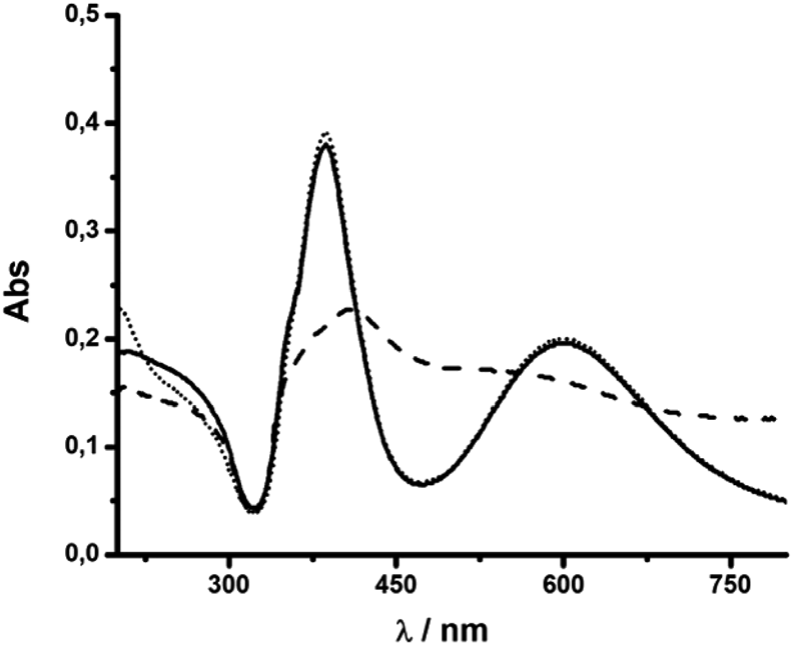
Stability of an AgNR sample followed by UV-visible spectroscopy at different times in days: 0 (^…^), 30 (—) and 90 (---).

**Fig. 5:**
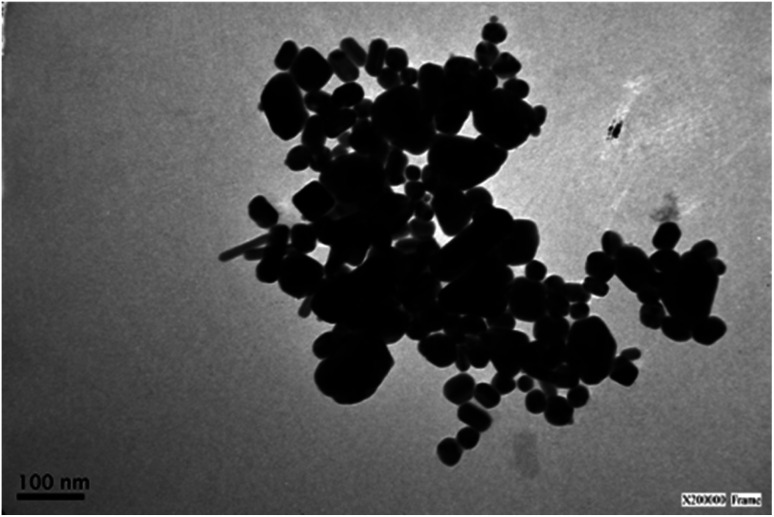
Representative TEM image of an AgNR sample after 3 months from the synthesis.

The stability of the mixture of nanoparticles was determined by following the UV-vis spectral changes for 90 days since the colloidal solution was prepared and stored. The absorption spectra observed in Fig. 4 show no changes at 30 days compared with the initial time, indicating the nanoparticles have not undergone any significant morphological or aggregation changes during a month. However, as the aging time increased (more than two months), the colloidal particles began settling at the vessels' bottom. They turned in green brownish, indicating particle aggregation and reshaping of the silver nanorods to quasi-spherical particles, attributed mainly to Ostwald ripening. The formation of large aggregates was corroborated by TEM observing particles larger than 100 nm in size (Fig. 5) and by the increased scattering above 700 nm and depleting the absorption around 400 nm observed in the UV-vis spectrum (Fig. 4).

### Estimation of nanoparticle concentration in a multi-shape and polydisperse sample

So far, the concentration of nanoparticles in polydisperse systems can be calculated when only spherical particles are present.^42^ When introducing a new morphology that presents a two-dimensional size distribution, as nanorods, it is necessary to adapt these models to calculate the volume of each particle and the probability of finding a particle of specific dimensions between all the NP. In that sense, we propose the MultiShapeC algorithm (eqn (3)) to estimate the concentration of nanoparticles in a colloidal system independently of shape and size distributions, even in mixtures, using TEM data. Table 1 shows a comparison between nanoparticle concentrations obtained using the MultiShapeC algorithm, which considers the mixture of spheres and rod particles with their corresponding polydispersity, and a method that only considers the average size of the particles.

**Table 1:** Comparison between concentrations of nanoparticles calculated using MultiShapeC *versus* the average size method

		Method	
		Average size^a^	MultiShapeC^b^
Nanosilver concentration (nM)	Mixture	8.5 (1.1)	0.370 (0.004)
	Rods	4.5 (0.6)	0.196 (0.002)
	Spheres	4.0 (0.5)	0.174 (0.002)

a Using [NP] = [*S*]/*N*; [*S*] is the Ag^+^ concentration and *N* is the ratio volume NP/volume Ag atom. In parenthesis, the error was estimated assuming the standard deviation from the mean of nanoparticle size to be proportional to the concentration.

b Using eqn (3). The error (in parenthesis) was estimated using error propagation.

**Fig. 6:**
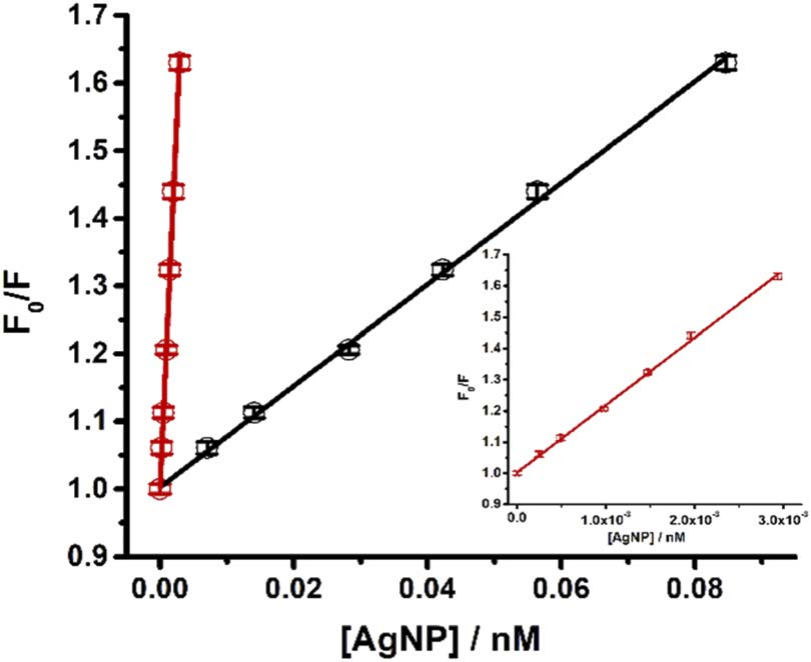
Stern–Volmer plots for the quenching of carbazole by AgNP. The concentration values in the *x*-axes were calculated using the average size method (black dots) or MultiShapeC (red dots). The slope corresponds to the Stern–Volmer constant. The inset shows a zoom on the red curve.

The quenching of the carbazole fluorescence emission by silver nanoparticles illustrates how the calculation of nanoparticle concentration in solution affects the study of the interaction between nanostructures and molecules (Fig. 6). This quenching responded to the Stern–Volmer equation.6
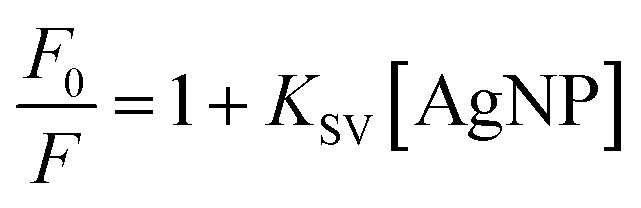
*K*_SV_ is the Stern–Volmer constant that relates the fluorescence emission's quenching to the nanomaterial's presence. The values for the *K*_SV_ (10^8^ M^−1^) determined using either the concentration estimated using MultiShapeC or the average size were (216 ± 4) and (7.5 ± 0.1), respectively. Notice that in this case, the NP concentration variation resulted in at least a 29-fold difference between the determined values of *K*_SV_. Therefore, it is expected that the impact would be more prominent for more polymorphic and polydisperse samples.

## Conclusions

In summary, we developed an algorithm, MultiShapeC, to calculate the concentration of polymorphic and polydisperse nanoparticle samples using TEM data. MultiShapeC was validated using silver particles containing rods and spheres as a case study. In addition, a seedless, surfactant-less, one-pot synthetic method was developed to produce samples containing nanorods (aspect ratio: 3) and nanospheres at a reproducible 1 : 1 proportion. Then, the impact of the nanoparticle concentration calculation on the determination of parameters like the Stern–Volmer constant was demonstrated. MultiShapeC renders a more realistic estimation of the actual colloidal nanoparticle concentrations, improving the determination of concentration-dependent parameters.

## Conflicts of interest

The authors declare that they do not have any conflicts of interest that could have influenced the outcomes of this work.

## Supplementary Material

RA-012-D2RA04078F-s001

RA-012-D2RA04078F-s002
