# Supplementary material for: MultiShapeC, an algorithm to assess concentration in multi-shape nanoparticle samples: nanosilver, a case study
Source: RSC Adv. 2022 Sep 20;12(41):26550–5. doi: 10.1039/d2ra04078f (PMC9486825; doi:10.1039/d2ra04078f)
Supplement: RA-012-D2RA04078F-s001 [file RA-012-D2RA04078F-s001.pdf]

Electronic Supplementary Material (ESI) for RSC Advances.
This journal is © The Royal Society of Chemistry 2022

Supplementary Material (ESI) for RSC Advances

MultiShapeC, an algorithm to assess concentration in multi-shape nanoparticle samples: Nanosilver, a case study

Rodrigo Nicolás Núñez,[a,b] Alicia Viviana Veglia[a,b] and Natalia Lorena Pacioni[a,b,*]

[a]Universidad Nacional de Córdoba, Facultad de Ciencias Químicas, Departamento de Química Orgánica. Haya de la Torre y Medina Allende s/n, X5000HUA, Ciudad Universitaria, Córdoba, Argentina.
[b]Consejo Nacional de Investigaciones Científicas y Técnicas (CONICET), INFIQC, Córdoba, Argentina.

*Corresponding author: nataliap@fcq.unc.edu.ar; n.lpacioni@unc.edu.ar

Contents

Page S1. This page
Page S2. Quantification of unreacted Ag^+
Page S2. Figure S1
Page S2. Figure S2
Page S3. Figure S3
Page S5. MultiShapeC tutorial
Page S6. References

Quantification of unreacted Ag^+

To establish the correct concentration of precursor metal ions to be entered into the MultiShapeC speadsheet, unreacted Ag^+ was determined using a spectrophotometric method described by Gonzalez-Fuenzalida that allows the in situ quantification of Ag^+ in presence of AgNP.[1] After the reaction between freshly prepared colloidal silver spiked with standard solutions of Ag^+ and 3,3',5,5'-tetrametihylbenzidine (TMB), the oxidized form of TMB (oxTMB), wich has a blue color, was obtained. UV-vis spectra were measured for each sample (Fig. S1) and the diference in absorbance signal at 656.0 nm and 543.0 nm ($\Delta Abs_{656-543}$) was recorded to perform calibration using the standard adition method (MOSA). The calibration curve is shown in Fig. S2. The slope of the calibration curve was (110 ± 2) 10^{-4} M^{-1} and the intercept was equal to zero, indicating that the amount of unreacted Ag^+ was less than the detection limit of the method. The detection limit (DL), calculated as DL= 3,3 Sb/m (Sb: standard deviation of the blanks; m: slope of the calibration curve), was equal to 3.5 μM.

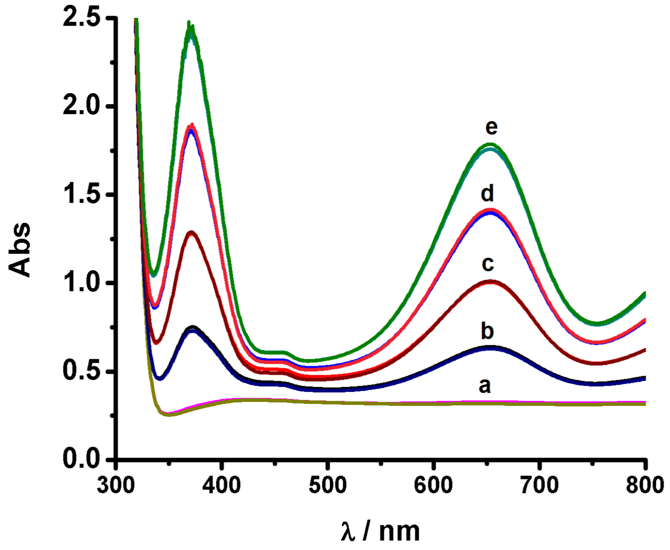

Figure S1. *UV-vis spectra of AgNR solutions spiked with increasing concentrations of* Ag^+ *0 - 100 μM (a-e) in presence of TMB.*

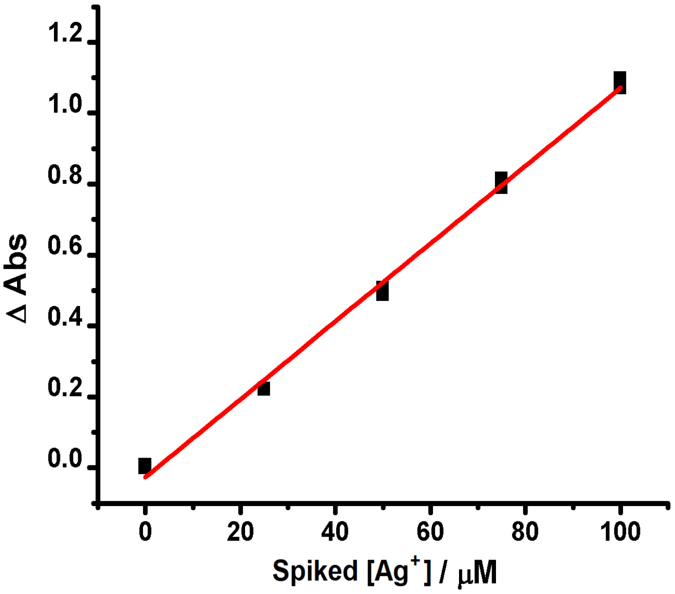

Figure S2. *MOSA calibration of spiked samples of AgNR by duplicate.*

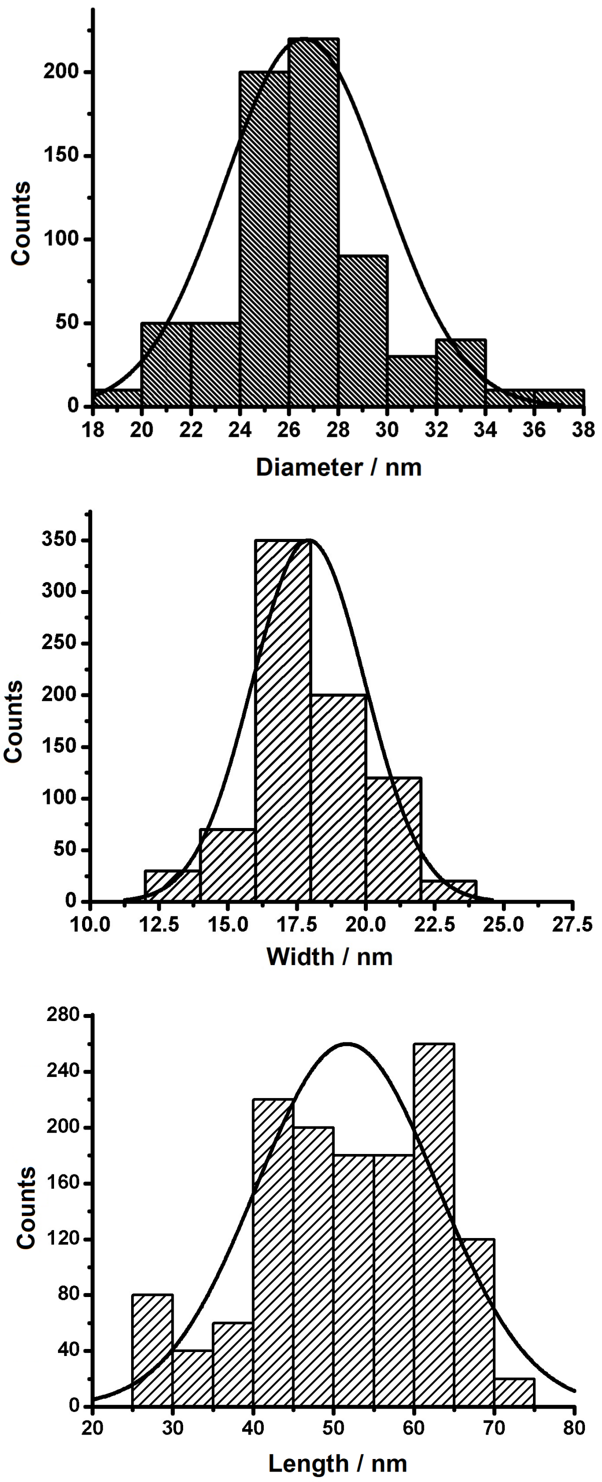

Figure S3. *Size distribution histograms of the synthesized silver nanospheres and nanorods. For spheres, particles have a mean diameter of 26 (3) nm. The rod-shaped particles have a mean length of 54 (9) nm and a width of 18 (2) nm. The standard deviation is shown in brackets.*

MultiShapeC Algorithm Tutorial

**All data is considered as nm, no use of exponential for sizes is required.*

1- NP's atoms identity must be indicated in the “NP metal” cell, colored light blue. Only silver and gold nanoparticles are considered. (Complete the cell with ag, Ag or silver; au, Au or gold, depending on your nanomaterial). This must be indicated in the page “General data input and results”.

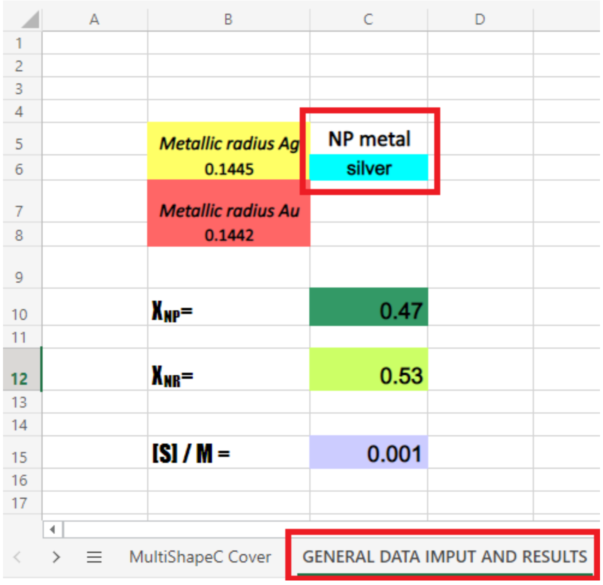

2- The spreadsheet only needs electron microscopy data to perform the calculations. For spherical particles, data must be entered into the page named SPHERES-DATA IMPUT, specifically in the column “Distribution percentages NP_i”. These percentages should be calculated for particles with the diameters indicated in the “$Diameter_i$” column. Diameters SHOULD NOT be edited, as MultiShapeC does calculations assuming 1 nm intervals. If there are no particles of a certain diameter in your sample, enter zeros.

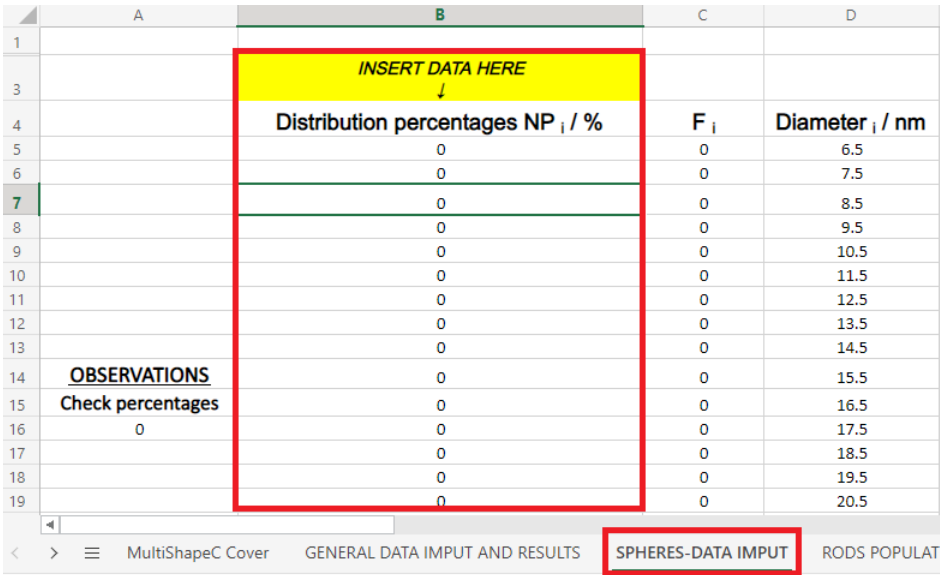

Anisotropic particles data is entered on the page named RODS POPULATION-DATA IMPUT, which is designed as a data matrix for particles of any combination of sizes (width and length). For rods, data must be the presence fraction, and not percentage as for spheres. Again, all calculations are performed considering 1 nm intervals. For this case, if no particle of a certain length and width is present, the cells can be left blank.

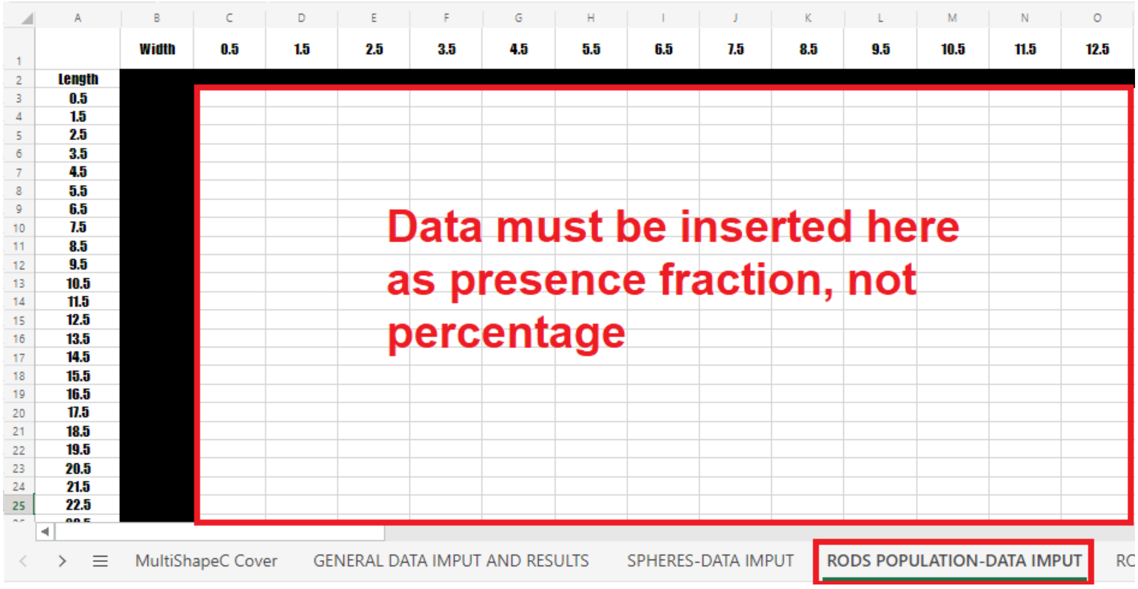

In both cases, data is related to the presence percentage of particles of a specific size relative to the total sample. As you insert your data into the corresponding column-pages, other MultiShapeC columns will be automatically filled.

3- NP and NR volumes are calculated considering the particles as spheres and cylindrical rods, respectively. This information is shown in the column V NP_i (SPHERES-DATA IMPUT) and in the page RODS-volumes. This information SHOULD NOT be edited.

4- Precursor ions – silver (I) and gold (III) - are considered spherical, and their volumes are calculated accordingly. The number of atoms that compose one NP is calculated as V NP_i/V_{atoms} in the column atoms per particle (SPHERES-DATA IMPUT) for spheres and in the page RODS-N° of atom per particle for rods.

5- The product S_i is calculated as the product between F_i and the N° of atoms per $particle_i$. For rods, this information is shown in the page RODS-CALCULATIONS. In all cases, the probabilistic interval (Δs for rods, Δa and Δb for rods) to find a particle of size "d" or rod of dimensions "w" and "l" is equal to the unity.

6- For spherical particles, the total sum is calculated by adding the first and the last cells with data of the column S_i, and the cells between the above, multiplied by 2. For example, as seen next, the sum is calculated adding the cells G9 (first data) and G16 (last data), and the cells G10 to G15 are incorporated into the calculation as 2*SUM(G10:G15). For rods, the total sum is calculated automatically from the auto-filled data in the page ROD SUMS.

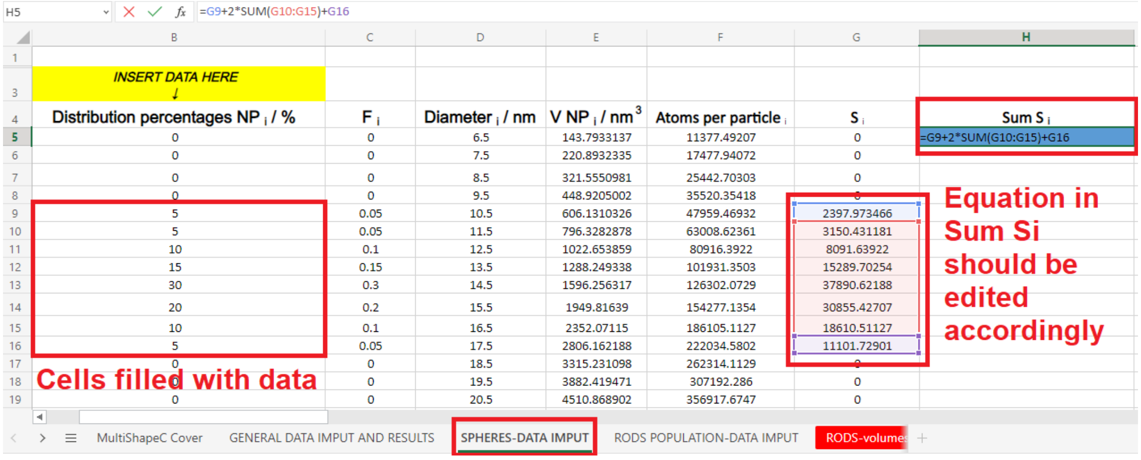

7- Relative proportion between spherical and rod-shaped nanoparticles must be indicated in the cells X_{NP} and X_{NR} (page GENERAL DATA IMPUT AND RESULTS). XNP + XNR must be equal to 1.

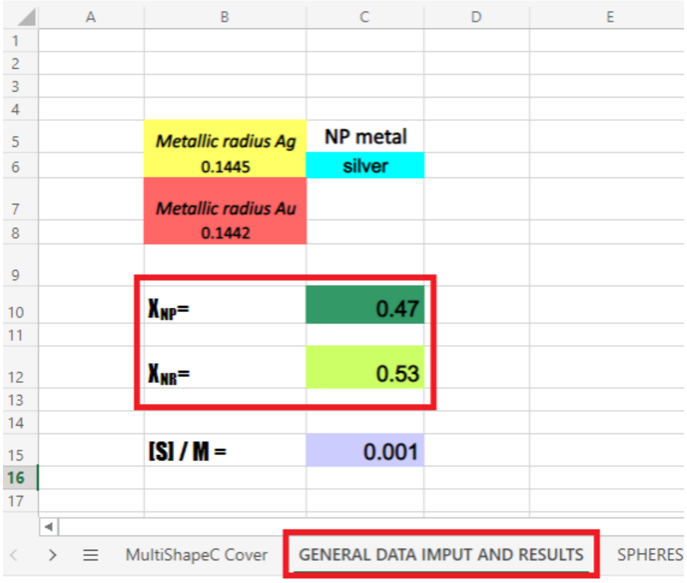

8- To perform the calculation of the total concentration of NP, MOLAR concentration of silver or gold cations must be informed in the cell [S] (page GENERAL DATA IMPUT AND RESULTS). Simultaneously, the concentration of spheres, rods and the total concentration are calculated, and the results are displayed in the cells [NP], [NR] and [NT], respectively. All calculated concentrations are molar (moles of particles per liter).

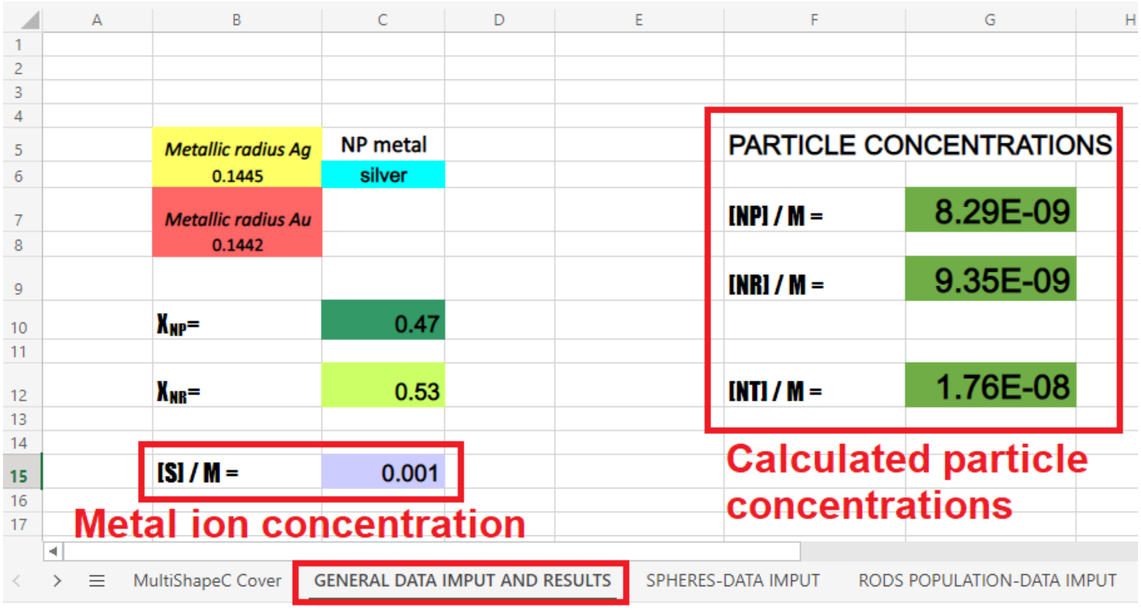

Reference

S1. González-Fuenzalida, R. A.; Moliner-Martínez, Y.; González-Béjar, M.; Molins-Legua, C.; Verdú-Andres, J.; Pérez-Prieto, J.; Campins-Falcó, P. In Situ Colorimetric Quantification of Silver Cations in the Presence of Silver Nanoparticles. Anal. Chem. 2013, 85 (21), 10013–10016. https://doi.org/10.1021/ac402822d.
